# Taliban's war on educating girls and women must end now: A call for global actions

**DOI:** 10.1002/puh2.80

**Published:** 2023-05-04

**Authors:** Shohra Qaderi, Adriana Viola Miranda, Goodness Ogeyi Odey, Shuaibu Saidu Musa, Leonard Thomas Sy Lim, Creuza Rachel Vicente, Joseph Christian Obnial, Aniekan Ekpenyong, Ahmed Said Abdou Elsayed Negida, Attaullah Ahmadi, Blaise Ntacyabukura, Brian Li Han Wong, Deborah Oluwaseun Shomuyiwa, Emery Manirambona, Isaac Olushola Ogunkola, Jaifred Christian F. Lopez, Julian M. A. Buban, Lila K. Chamlagai, Nelson Ashinedu Ukor, Paolo Miguel Manalang Vicerra, Yusuff Adebayo Adebisi, Yasir Ahmed Mohammed Elhadi, Don Eliseo Lucero‐Prisno

**Affiliations:** ^1^ Maternal‐Fetal Care Center, Boston Children’s Hospital Harvard Medical School Massachusetts Boston USA; ^2^ Global Health Focus (GHF) Asia Bandung Indonesia; ^3^ Department of Public Health College of Medical Sciences, University of Calabar Calabar Nigeria; ^4^ Department of Nursing Science Ahmadu Bello University Zaria Nigeria; ^5^ College of Medicine University of the Philippines Manila Manila Philippines; ^6^ Department of Social Medicine Federal University of Espírito Santo Espírito Santo Vitória Brazil; ^7^ Faculty of Medicine and Surgery University of Santo Tomas Manila Philippines; ^8^ Faculty of Health, Social Care and Medicine Edge Hill University Ormskirk UK; ^9^ Department of Neurology Virginia Commonwealth University Virginia Richmond USA; ^10^ Department of Epidemiology and Biostatistics École des Hautes Études en Santé Publique Paris France; ^11^ Department of Global Public Health Karolinska Institute Solna Sweden; ^12^ Department of International Health Care and Public Health Research Institute (CAPHRI), Maastricht University Maastricht The Netherlands; ^13^ Association of Schools of Public Health in the European Region (ASPHER) Brussels Belgium; ^14^ Faculty of Pharmacy University of Lagos Lagos Nigeria; ^15^ College of Medicine and Health Sciences University of Rwanda Kigali Rwanda; ^16^ Department of Population Health Sciences Duke University North Carolina Durham USA; ^17^ College of Medicine University of the Philippines Manila Philippines; ^18^ School of Public Health Brown University Providence Rhode Island USA; ^19^ Faculty of Pharmacy University of Port Harcourt Port Harcourt Nigeria; ^20^ Asian Demographic Research Institute Shanghai University Shanghai China; ^21^ Nuffield Department of Population Health University of Oxford Oxford UK; ^22^ Department of Public Health Sudanese Medical Research Association Khartoum Sudan; ^23^ Department of Global Health and Development London School of Hygiene and Tropical Medicine London UK; ^24^ Faculty of Management and Development Studies University of the Philippines Open University Los Baños Laguna Philippines; ^25^ Faculty of Public Health Mahidol University Bangkok Thailand

The Taliban's decision to ban women's and girl's education in Afghanistan paints a bleak picture of the future of Afghan women [1]. It is a direct violation of the Universal Declaration of Human Rights (UDHR), which states that everyone has the right to education [2]. Deprivation of education is known to negatively affect other human rights, such as the rights to health and freedom. We, as health journal editors from across six continents, call for urgent international actions to restore justice and protect human rights in Afghanistan.

The Taliban's takeover in August 2021 added massive pressure to the already overwhelmed health system of Afghanistan [3]. Afghan women and girls have been disproportionately affected, as the Taliban adhere to an ultra‐fundamentalist interpretation of Islam that severely restricts women's freedom of movement and access to healthcare, education, and employment. Although the Taliban have repeatedly claimed that they would protect the rights of girls and women, they have in fact done the opposite. Some of their most drastic restrictions have been around education. In March 2022, the Taliban banned girls from returning to secondary schools [5]. This was followed by another decision in December 2022 to indefinitely ban women and girls from pursuing higher education and work [1]. Years of progress were reversed overnight, with Afghanistan once again becoming the only country in the world that actively prevents girls from receiving an education.

The education crisis has had a huge impact on the social determinants of health across the country, leading to devastating health outcomes for Afghan women [4]. Even before the ban on education, Afghanistan's health indicators had already been rapidly deteriorating since the Taliban takeover. A United Nations Women survey reported that in 2022, only 10% of women could cover their basic health needs, compared to 23% of men [5]. This is a major setback compared to the 90% coverage of basic health services that the Afghan population had just prior to the takeover [6]. Another survey reported staff shortages, particularly because the Taliban only allow women to be attended by female healthcare workers. The maternal mortality rate, after decades of significant reductions, is projected to be on the rise [7].

The deprivation of education and the increasing restrictions that act to keep women confined to their homes impact every aspect of their well‐being, including mental and physical health, food insecurity, immunization status, and access to medical care [5, 6, 7, 8, 9]. Prior to and during the first year of the Taliban's regime, school‐based public health interventions had been vital for reaching Afghan girls. For instance, the United Nations reported that between August 2021 and 2022, 272,386 adolescent girls received iron and folic acid supplementation in schools [10].

Schools had also provided safe spaces for Afghan women. They reduced the risk of exploitation, abuse, and child and forced marriage [10, 11]. In 2016, Afghan government data reported that girls deprived of education had their risk of entering child marriage increased threefold compared to girls who completed at least secondary education [11]. As school closures become more rampant, Afghan women become deprived of their right to health and freedom from violence. The education ban is also denying them and the whole nation of better socioeconomic conditions, which are inextricably linked to improving national health status. The United Nations Children's Fund (UNICEF) estimated that in the last 12 months, the ban cost Afghanistan at least US$500 million [10]. Furthermore, the ban also cuts the access of female healthcare students to education, exacerbating the shortage of female healthcare workers [7, 11]. This is detrimental to the health status of women, who are now unable to seek care from both male healthcare workers and the ever decreasing number of female health workers.

The grim reality that is unfolding in Afghanistan has a striking resemblance with the reality of the Taliban's first rule in the 1990s, which also banned girls’ education. At that time, persistent conflicts, deprivation of education, and increasing poverty rates led to the collapse of Afghanistan's health system and provided almost no access to health services for women. This was partly due to the severe international sanctions imposed on the Taliban regime. The situation improved during the decades of post‐Taliban rule, which saw significant international support through financial aid and other development support [12]. This highlights the importance of international and collective actions. Protecting human rights and improving living conditions should be conducted at the global level due to its huge impact and moral reasons‐to foster long lasting peace, humans must not leave others behind. Actions against the Afghan crisis, while not directly affecting other countries, are a testament to the belief that human rights should be universal, irrespective of one's gender, belief, ethnicity, or nationality.

As history repeats itself with the resurgence of the Taliban regime, the international community is once again imposing sanctions on the Taliban. Although these efforts may be successful at pressuring the Taliban government, their severe impact on the ordinary civilians, including women, should also be considered. Coupled with the Taliban's active deprivation of women's rights, sanction‐related poverty has worsened women's living conditions. In addition, the complexity of operating humanitarian efforts amidst international sanctions continues to limit access to basic services [13]. Balancing these political and humanitarian efforts should be the priority of the international community.

Now more than ever, the world has become more connected. This calls for urgent actions and increased pressure from the international community to end the Taliban's restrictions on the right of Afghan women and girls to quality education. Recognition, aid, and legitimization of the Taliban by the international community should be hinged upon visible proof of inclusivity, respect for human rights, and people‐centered governance, beginning with girls being able to access equitable and quality education across all levels. There is a need for dialogue with the Taliban on the importance of women and girls education for national growth and socioeconomic development, as well as strong condemnation of the current school ban. This engagement is best led by Islamic scholars, influential personalities, and religious leaders, who are well poised to advocate for the prioritization of equal and quality education in the Islamic context. Although the current ban continues, international development organizations in Afghanistan should explore and provide alternative educational support to girls, which bring them at pace with the boys in school [14].

Ending the Taliban's war against women, such as the education ban, requires global actions. Everyone, including Afghan women, is entitled to the protection of their basic needs and rights. The actions of the global community will not only affect the current generation but also Afghan generations to come.

## CONFLICT OF INTEREST STATEMENT

All authors are editorial board members of the journal. They were excluded and blinded from all stages of the peer review of this manuscript.

## FUNDING INFORMATION

There is no funding for the development of this paper.

## ETHICS STATEMENT

There is no need for ethical approval.

## Data Availability

No database or primary data was used in writing the paper.
